# *Tropheryma whipplei* in the stool samples of children with acute diarrhea: a study from Tehran, Iran

**DOI:** 10.1186/s12879-022-07198-5

**Published:** 2022-02-27

**Authors:** Shirin Sayyahfar, Mina Latifian, Parisa Esmaeili, Neda Baseri, Fahimeh Bagheri Amiri, Bita Bakhshi, Abdoulreza Esteghamati, Saber Esmaeili

**Affiliations:** 1 Research Center of Pediatric Infectious Diseases, Institute of Immunology and Infectious Diseases, Iran University of Medical Sciences, Tehran, Iran; 2 National Reference Laboratory for Plague, Tularemia and Q Fever, Research Centre for Emerging and Reemerging Infectious Diseases, Pasteur Institute of Iran, Akanlu, Kabudar Ahang, Hamadan, Iran; 3 Department of Epidemiology and Biostatistics, Research Centre for Emerging and Reemerging Infectious Diseases, Pasteur Institute of Iran, Tehran, Iran; 4 Department of Bacteriology, Faculty of Medical Sciences, Tarbiat Modares University, Tehran, Iran

**Keywords:** *Tropheryma whipplei*, Diarrheas, Whipple's Disease, Gastrointestinal Disease

## Abstract

**Background:**

Recently, *Tropheryma whipplei* has been suggested as one of the causative agents of diarrhea among children worldwide. Limited data is available on the prevalence of *T. whipplei* among children with diarrhea in most countries such as Iran. This study was conducted to evaluate the prevalence of *T. whipplei* in children with acute diarrhea in Iran.

**Methods:**

In this study, the stool samples were collected from 130 children under 10 years old with acute diarrhea from children's hospitals in Tehran city. Genomic DNA was extracted from stool samples and was tested for the presence of DNA of *T. whipplei* using the SYBR Green Real-time PCR method. Positive *T. whipplei* samples were finally confirmed by PCR Product sequencing.

**Results:**

The mean age of participants was 32.5 months, and 54.6% of children were female. Using the SYBR Green Real-time PCR, 9.23% (12/130) of samples were positive for *T. whipplei*, which were confirmed by sequencing. 66.67% of positive cases were males. The duration of diarrhea in infected children with *T. whipplei* (83.3%) was significantly longer (OR: 5.93, 95% CI 1.24–28.22) compared to children with negative results (45.8%). Other demographic factors and clinical signs had not a statistically significant relationship with *T. whipplei* infection.

**Conclusions:**

In this study, *T. whipplei* was detected in stool samples of children with acute diarrhea. The results indicated that *T. whipplei* could be associated with childhood diarrhea in Iran. The health care system and physicians should be aware of the presence of *T. whipplei* infection in Iran, especially in childhood diarrhea.

**Supplementary Information:**

The online version contains supplementary material available at 10.1186/s12879-022-07198-5.

## Background

*Tropheryma whipplei* is a road shape Gram-positive bacterium that causes Whipple’s disease. The human intestine is the only known natural site of this microorganism. After the colonization of *T. whipplei* in the intestinal mucosa, it is taken up by macrophages cells and then replicates inside them [1].

*T. whipplei* has been detected in various biological specimens of infected people, including saliva, urine, blood, heart valve, myocardium, synovial fluid, skeletal muscle, feces, skin, lymph nodes, lung, bronchoalveolar fluid, stomach, spleen, liver, larynx, small intestine, colon, maxillary sinus, cerebrospinal fluid, and aqueous humor. *T. whipple*i can persist in vectors for several years, and asymptomatic carriers can become a large reservoir for infecting others [2]. Whipple’s disease is mainly transmitted by the oral–oral or fecal–oral routes. A high prevalence of this disease was reported among people with poor quality health, sewage workers, and homeless people [3, 4]. There are four forms of *T. whipplei* infection in humans, including classic Whipple disease, chronic localized infection, acute infection, and asymptomatic form [2]. The classic form of the Whipple disease is most common in men. Arthralgia, diarrhea, fatty diarrhea, loss of weight, lymphadenopathy, abdominal pain, hypoalbuminemia, and anemia are often observed as clinical symptoms of this form of Whipple's disease [5]. All the organs, in particular the eyes, heart, and central nervous system can be involved at the end stage of the disease. Local infection with *T. whipplei* is often without gastrointestinal involvement. However, it can be associated with encephalopathy, endocarditis, respiratory problems, lymphadenopathy, osteoarticular, and uveitis [2]. Recently, it was reported that *T. whipplei* caused acute infections, including gastroenteritis, bacteremia, or pneumonia [1]. Although diarrhea is one of the symptoms of the chronic, classic, and acute forms of *T. whipplei* infection, diagnosis is often difficult because there are no specific endoscopic findings, and this bacterium is not detectable by routine stool culture. The histopathology and PCR methods are the most well-known *T. whipplei* diagnostic approaches [6, 7]. However, histopathological examination of the biopsy specimen is not commonly used for screening purposes because it is a costly and invasive method. In contrast, PCR can be preferred to identify *T. whipplei* in various specimens (e.g., stool, mucosa, and fluids), and it is a fast, specific, sensitive, and cost-effective method [2]. In addition, transmission electron microscopy is a helpful diagnostic tool for identifying both living and dead bacteria after therapy, when available [8]. Early diagnosis can help to treatment and eradication of *T. whipplei* in patients. However, misdiagnosis or improper treatment, which especially occurs in patients without the classic symptoms, can lead to lethal outcomes or irreversible neurological damage. Sometimes, patients with early symptoms may also be hard to treat due to the presence of bacteria in an inaccessible niche for the antibiotic, antibiotic resistance, or reinfection in late relapses after antibiotic treatment. Where combination therapy with antibiotics is helpful, immunosuppressive treatment can be a risk factor for displaying or aggravating Whipple's disease [2, 9, 10]. Diarrhea in children is a significant health threat in developing countries that is caused by various microorganisms. Recent studies in many countries have shown that *T. whipplei* is one of the infectious causes of diarrhea in children. The results of these studies showed that the prevalence of *T. whipplei* was more in the stool of children with diarrhea than healthy children as a control group. For instance, using the qPCR method, stool prevalence of *T.whipplei* was 27.5% among children with diarrhea from Ghana. That case group carried *T.whipplei* in their stool twice as frequently as controls without diarrhea [11]. In another case–control study, *T. whipplei* was identified in 15% of stool samples collected from children with gastroenteritis using the qPCR method. In addition, *T. whipplei* antibody was reported in the case group. None of the control cases were positive for the presence of bacterium or antibody. Interestingly, *T. whipplei* was not detected in the stool samples of children after recovering from diarrhea [12]. Therefore, *T. whipplei* should be considered as one of the potential causes of diarrhea in children. Since clinical symptoms are not sufficient for *T. whipplei* diarrhea diagnostic, molecular tests are essential to confirm this disease. In Iran, similar to most countries of the world, there is no comprehensive survey on the prevalence of *T. whipplei*, particularly in children with diarrhea. Therefore, the present study was performed to evaluate the presence of *T. whipplei* in stool samples of children with acute diarrhea using molecular methods.

## Materials and methods

### Sample collection

One hundred and thirty children under 10 years old with acute diarrhea who were referred to three hospitals in Tehran province (Bahrami Hospital, Mofid Hospital, and Children's Medical Center) from May 2018 to November 2018 were included in this cross-sectional study. The questionnaire form containing demographic, clinical, and laboratory information was completed at admission time.

Diarrhea stool samples were obtained from included children with acute diarrhea. Sampling was performed under an identical process and according to the hospital guidelines and regulations. A healthcare professional explained to the parents how to collect the stool of their children. Briefly, fresh diarrhea stools (10 gr) were collected inside a completely clean, dry, and sterile container with a tight screw lid. Urine was not mixed up with stool.

Stool samples were transferred to the laboratory under the cold chain conditions immediately. Future analysis was performed as soon as possible; otherwise, specimens were sore in −20 until the investigation.

### DNA extraction

Genomic DNA was extracted from stool samples using the QIAamp DNA Stool Minikit (Qiagen, Germany), according to the manufacturer's recommendation. 220 mg of each stool sample was used for DNA extraction.

To determine quality control of extracted DNA from stool samples as well as control DNA, the DNA absorbance at 260 nm and 280 nm was absorbed using a microplate spectrophotometer (Epoch bioTek, USA). Absorbance ratios of 260/280 nm around 1.8 were considered as good quality. Higher or lower values were considered as RNA or protein contamination. In addition, extracted DNAs were be run on gel agarose to determine quality.

The extracted DNA was stored at −20 ℃ until the future test.

### Molecular detection of *Tropheryma whipplei*

All extracted DNA samples were tested using SYBR Green quantitative real-time PCR (qPCR) for the diagnosis of the WiSP protein family gene of *T. whipplei*.

Tws1F (5′-AGAGAGATGGGGTGCAGGAC-3′) and Tws1R (5′-AGCCTTTGCCAGACAGACAC-3) were used as forward and reverse primers (Metabion international, Germany), respectively. The qPCR mixture included 10 μl RealQ Plus 2 × Master Mix Green Low ROX™ (Ampliqon, Denmark), 1 μl from each primer (900 nmol), and 4 μl of extracted DNA (11). The mixture was reached to 20 μl with sterile distilled water. *T. whipplei* DNA was provided from IRCCS Sacro Cuore Don Calabria Hospital in Italy and applied as the positive control. Sterile distilled water was used as the negative control. The mixture was run in the Corbett 6000 Rotor-Gene system (Corbett, Victoria, Australia) under the following temperature program: Initial denaturation step at 95 °C for 15 min, and then 40 cycles of 95 °C for 15 s, 30 s at 60 °C, and 72 °C for 30 s. After amplification, the melting protocol was followed by raising the temperature slowly from 65 to 97 °C to differentiate the specific and non-specific amplified amplicons. The samples with suitable amplification and melting (80 ± 0.5 °C) curves were considered positive for *T. whipplei*.

To confirm *T. whipplei* positive samples, PCR products were sequenced using the Sanger sequencing method. Finally, The nucleotide sequences were blasted in GenBank (https://blast.ncbi.nlm.nih.gov/Blast.cgi) and compared with the deposited *wips* genes of *T. whipplei* in the GenBank.

### Statistical analysis

Statistical analysis was performed using SPSS software version 18 (SPSS Inc., Chicago, IL, USA) for the data of this study. Chi-squared and Fisher exact tests were used to compare the variables. A p-value less than 0.05 was considered statistically significant.

## Results

One hundred and thirty children with acute diarrhea were included in this study. The mean age of children was 32.5 ± 31.6 months; 45.4% were male, and 54.6% were female. The mean frequency of diarrhea was four times a day. The mean duration of diarrhea was 14.6 days, and the median was 3 days. Also, 42.3% of children had a fever, and 5.38% had an underlying disease (Table 1).

**Table 1:** Demographic and clinical characteristics of children with acute diarrhea and their relationship to the prevalence of *T. whipplei*

	Negative *T. whipplei* N = 118	Positive *T. whipplei* N = 12	P value	OR (95% CI)
Gender (male)	51 (43.2)	8 (66.7)	0.12	2.63 (0.75–9.21)
Age median ≥ 32.9 month	41 (34.7)	5 (41.7)	0.75	1.34 (0.40–4.49)
Median times of diarrhea (days): ≥ 4 times	63 (53.4)	8 (66.7)	0.38	1.75 (0.50–6.11)
Median times of diarrhea (days) ≥ 3	54 (45.8)	10 (83.3)	0.01*	5.93 (1.24–28.22)
Fever	51 (43.2)	4 (33.3)	0.51	0.66 (0.19–2.30)
Stomachache	29 (24.6)	5 (41.7)	0.20	2.19 (0.65–7.44)
Cramps	34 (28.8)	1 (8.3)	0.18	0.91 (0.83–1.00)
Headache	4 (3.4)	1 (8.3)	0.39	2.59 (0.27–25.26)
Vomiting	29 (24.6)	4 (33.3)	0.50	1.53 (0.43–5.47)
History of contact with animal	3 (2.5)	0 (0.0)	0.99	–
Family history of diarrhea	10 (8.5)	0 (0.0)	0.60	–
Travel history	6 (5.1)	0 (0.0)	0.99	–
Underlying disease	5 (4.2)	2 (16.7)	0.13	4.52 (0.78–26.34)
Mucus in stool	37 (31.4)	4 (33.3)	0.99	1.10 (0.31–3.87)
Blood in stool	11 (9.3)	1 (8.3)	0.99	0.88 (0.10–7.51)
Pus in the stool	10 (8.5)	1 (8.3)	0.99	0.98 (0.12–8.41)
WBC in stool	24 (20.3)	3 (25.0)	0.71	1.31 (0.33–5.20)

The symbol (*) indicates statistically significant between children with positive *T. whipplei* and children without *T. whipplei* infection. A p value < 0.05 was considered significant

Extracted DNAs had good quality in 260/280 measurement (ratios around 1.8) and on gel agarose. Using the SYBR Green qPCR method, *T. whipplei* was detected in 9.2% (12/130). Then, the positive results were sequenced to confirm. The amplification and melt curves obtained from SYBR Green real-time PCR for *Tropheryma whipplei* detection based on the *wips* gene are shown in the Additional file 1: Figure S1.

Among patients infected with *T. whipplei*, 66.7% were male, 58.3% were less than 32 months, and 83.3% had diarrhea for more than 3 days. Also, 66.7% of positive children had diarrhea more than four times a day. People with positive results (83.3%) had significantly more diarrheal days than people with negative results (45.8%) (OR: 5.93, 95% CI 1.24–28.22). No correlation was found between positive results and other demographic factors and clinical signs (Table 1).

## Discussion

Although *T. whipplei* colonization is often asymptomatic and especially common in adults, the carriers can transmit the microorganism to healthy people. Diarrhea is one of the main manifestations of *T. whipplei* infection in humans. Gastroenteritis occurs in both classic and acute forms of Whipple's disease [2]. *T. whipplei* is known as a causative agent of diarrhea in children in all developed and developing countries. Therefore, investigation of acute diarrhea associated with *T. whipplei* in Iran is one of the health priorities that physicians and health officials should be aware of the role of this bacterium in various syndromes, especially acute diarrhea in Iranian children. In the present cross-sectional study, 9.23% of collected stool samples of children with acute diarrhea referred to three hospitals in Tehran city (in Iran) during 2018 were positive for *T. whipplei* using the qPCR method. As far as we know, except for a recently reported study, there have not been any studies on *T. whipplei* in Iran so far. In that study, 10% of immunocompromised children in a province (Qom) in Iran had *T. whipplei* DNA (using qPCR) in their stool samples [13]. Since *T. whipplei* is a hard-growing bacterium, bacterial culture requires a long incubation time that is not available in many laboratories. Therefore, molecular methods are a preferred method to identify *T. whipplei* infection in patients [14, 15]. For instance, qPCR is a beneficial method for first-line screening of *T. whipplei* in stool specimens [16].

This study shows that the prevalence of *T. whipplei* is much lower than similar studies conducted in other countries, including Ghana 27.5% [11], France 15% [12], Gabon 40% [17], Laos 48% [18], and Senegal 31.2% [19]. Various factors, such as differences in health care systems and climatic conditions, could be involved in incidence variations between different regions in the world. The prevalence of diarrhea caused by *T. whipplei* in children is significantly higher in tropical regions, particularly in countries with low levels of health [2, 5]. In the present study, there was no association between *T. whipplei* infection with history of travel, contact with animals, family history of diarrhea, and a set of clinical and laboratory findings. However, diarrhea for ≥ 3 days was observed among 83.3% of children with *T. whipplei* infection. In contrast, only 45.8% of children with negative *T. whipplei* experienced prolonged diarrhea. According to our results, it seems that *T. whipplei* might cause prolonged diarrhea compared to previously known bacterial agents causing diarrhea.

Since *T. whipplei* is an unknown causative agent of acute diarrhea in children among physicians and laboratories in Iran, our results, even with this reported prevalence, can be a warning to inform physicians and laboratories of the existence of this disease in Iran. Considering that *T. whipplei* may colonize all ages asymptomatically, future epidemiological studies are required to assay the association of *T. whipplei* with acute diarrhea in children in different parts of the world, including Iran. In addition, diagnosis, control, and treatment of *T. whipplei* should be considered by health systems in Iran and even other countries.

One of the limitations of the study is the small number of samples. Future studies among a larger population with different demographic and clinical characteristics can determine comprehensive knowledge on the frequency of *T. whipplei* in patients with diarrhea. In addition, this study did not include a control group (children without diarrhea) which made result interpretation more difficult. Another limitation was that we did not measure possible antibodies produced against *T. whipplei.* Furthermore, no other clinical microbiology diagnosis was performed to identify other enteric pathogens in positive samples of *T. whipplei*. These data could help to interpret of obtaining results in the present studies.

## Conclusions

In this study, the stool prevalence of *T. whipplei* among children with acute diarrhea in Iran was 9.23% using the qPCR method. Significantly, children with *T. whipplei* infectious diarrhea had prolonged diarrhea than children without *T. whipplei* infection (83.3% vs. 45.8%). Therefore, *T. whipplei* could be a potential cause of acute diarrhea in children in Iran, and physicians and laboratories should be aware of the presence of this disease in Iran. However, comprehensive studies are required to evaluate the epidemiology and pathogenicity of *T. whipplei* in pediatric diarrhea in Iran. This study showed the importance of qPCR in the diagnosis of *T. whipplei* infection as a possible cause of diarrhea in children. Therefore, molecular diagnosis of *T. whipplei* is recommended, especially when diarrhea lasts more than 3–4 days.

## Supplementary Information

**Additional file 1: Figure S1.** Representative the amplification plot (A) and melting curve (B) of SYBR Green real-time PCR for *Tropheryma whipplei* detection based on the *wips* gene in specimens. Positive and negative controls were run in all runs. A) The cycle number is shown on the x-axis and change in fluorescent intensity is shown on the y-axis. B) Positive control and samples have a melt pick in 80 ± 0.5 °C in melting curves.

## Data Availability

The datasets used and/or analyzed during the current study are available from the corresponding author on reasonable request.

## Abbreviations

*T. whipplei*: *Tropheryma whipplei*
qPCR: Quantitative real-time PCR
