# Supplementary material for: Tropheryma whipplei in the stool samples of children with acute diarrhea: a study from Tehran, Iran
Source: BMC Infect Dis. 2022 Feb 27;22:193. doi: 10.1186/s12879-022-07198-5 (PMC8883655; doi:10.1186/s12879-022-07198-5)
Supplement: Supplementary file 1 — Additional file 1: Figure S1. Representative the amplification plot (A) and melting curve (B) of SYBR Green real-time PCR for Tropheryma whipplei detection based on the wips gene in specimens. Positive and negative controls were run in all runs. A) The cycle number is shown on the x-axis and change in fluorescent intensity is shown on the y-axis. B) Positive control and samples have a melt pick in 80 ± 0.5 °C in melting curves. [file 12879_2022_7198_MOESM1_ESM.docx]

**Additional file 1**

*Tropheryma whipplei* in the stool samples of children with acute diarrhea: A study from Tehran, Iran

Shirin Sayyahfar1, Mina Latifian2,3, Parisa Esmaeili2,3, Neda Baseri2,3, Fahimeh Bagheri Amiri3, Bita Bakhshi4, Abdoulreza Esteghamati1, Saber Esmaeili2,3*

1. Research Center of Pediatric Infectious Diseases, Institute of Immunology and Infectious Diseases, Iran University of Medical Sciences, Tehran, Iran.
2. National Reference Laboratory for Plague, Tularemia and Q fever, Research Centre for Emerging and Reemerging infectious diseases, Pasteur Institute of Iran, Akanlu, Kabudar Ahang, Hamadan, Iran.
3. Department of Epidemiology and Biostatistics, Research Centre for Emerging and Reemerging infectious diseases, Pasteur Institute of Iran, Tehran, Iran.
4. Department of Bacteriology, Faculty of Medical Sciences, Tarbiat Modares University, Tehran, Iran

*Correspondence should be addressed to Saber Esmaeili; dr.saberesmaeili@gmail.com


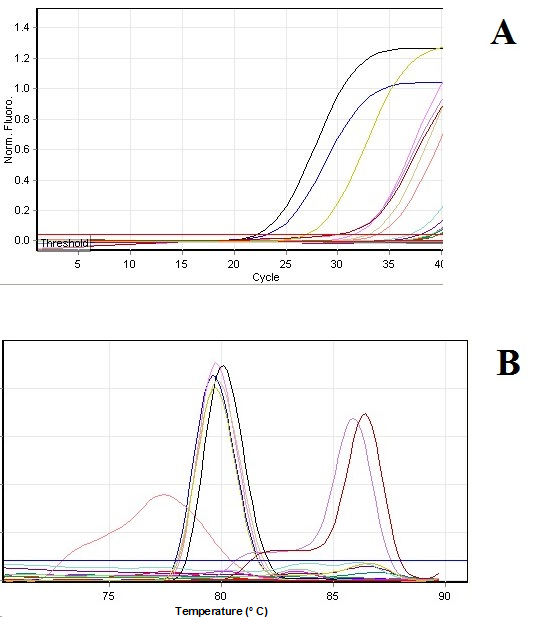


Fig. S1. Representative the amplification plot (A) and melting curve (B) of SYBR Green real-time PCR for *Tropheryma whipplei* detection based on the wips gene in specimens. Positive and negative controls were run in all runs. A) The cycle number is shown on the x-axis and change in fluorescent intensity is shown on the y-axis. B) Positive control and samples have a melt pick in 80 ± 0.5 ̊C in melting curves.
